# Impact of early versus delayed mobilization after arthroscopic rotator cuff repair in small and medium-sized tears: a prospective randomized study

**DOI:** 10.1186/s13018-026-07284-3

**Published:** 2026-11-10

**Authors:** Mohamed Rabie Abdallah, Ahmed Shaban Hassan, Mohamed Abdelal

**Affiliations:** 1 Orthopedics Department, Faculty of Medicine, Helwan University, Helwan, Cairo, 11795 Egypt; 2 National Institute for the Musculoskeletal and Nervous System, Cairo, Egypt

**Keywords:** Early mobilization, Constant–Murley, Range of motion, Sized tears, Adult patients

## Abstract

**Purpose:**

To compare early versus delayed mobilization following arthroscopic single-row knotless rotator cuff repair for small and medium-sized tears, performed in the beach-chair position without the use of an abduction sling.

**Methods:**

A prospective randomized study was conducted on 70 patients aged 20–70 years with small and medium-sized full-thickness supraspinatus tears repaired using single-row knotless suture anchors (one or two depending on tear size). Patients were assigned to early mobilization (EPM, *n* = 35) or delayed mobilization (DPM, *n* = 35). Outcomes were assessed using range of motion (ROM), pain (VAS), Constant–Murley and UCLA scores, and MRI tendon integrity at 12 months.

**Results:**

EPM patients showed faster ROM recovery at 3 months (mean 130° vs. 115°; *p* = 0.02), but one re-tear (2.8%) occurred in this group compared to DPM, where no re-tears occurred. At 12 months, both groups achieved comparable functional and pain outcomes.

**Conclusion:**

Early mobilization accelerates short-term ROM recovery, whereas both early and delayed protocols result in comparable functional and pain outcomes by 12 months. Given the limited number of retear events and the absence of a prespecified tear size subgroup analysis, no definitive recommendation can be made regarding a differential mobilization strategy according to tear size.

**Graphical Abstract:**

**Figure:**
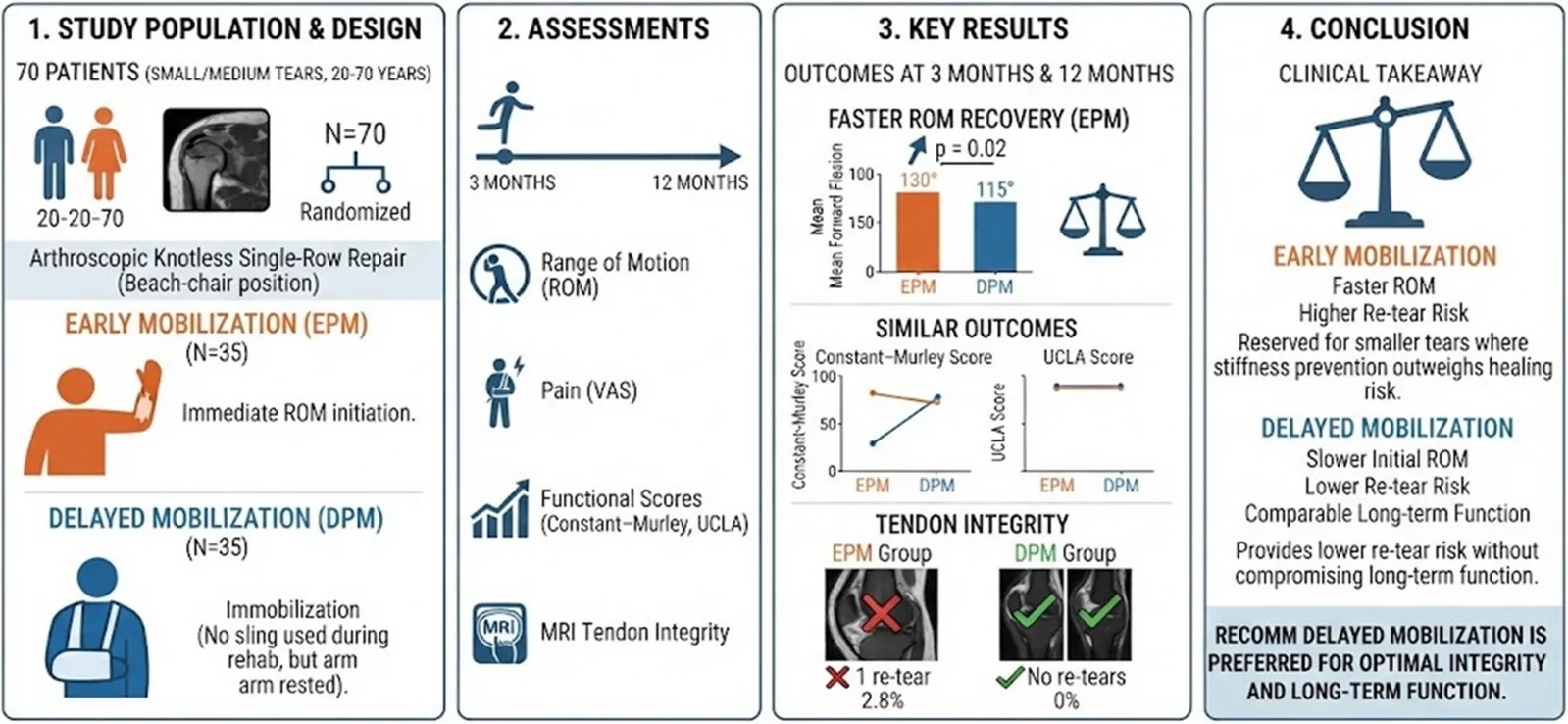
The graphical abstract illustrates a prospective randomized study comparing early mobilization (EPM) versus delayed mobilization (DPM) in 70 patients following arthroscopic single-row knotless rotator cuff repair. Structured across four distinct panels, it outlines the patient cohort, clinical assessment metrics, empirical results, and final conclusions. The findings demonstrate that while the EPM group achieved a significantly faster range of motion recovery in 3 months (130 versus 115, *p* = 0.02), it also experienced a higher structural re-tear rate (2.8% versus 0%) compared to the DPM group.

## Key messages

- Early mobilization speeds up short-term range of motion (at 3 months), but delayed mobilization significantly lowers the risk of tendon re-tears.

- Both approaches achieve the same excellent pain relief and functional recovery by 12 months.

## Contributions to the literature

- Provides highly specific data for small and medium-sized tears repaired with single-row knotless anchors, separating them from broader studies that include massive tears.
- Clearly quantifies the clinical trade-off between early joint stiffness prevention and structural tendon healing.

## Response impact (clinical impact)

- Gives surgeons and physical therapists clear evidence to safely default to delayed mobilization to protect the repair without compromising long-term function.
- Enables personalized rehabilitation plans, reserving early mobilization only for patients where preventing joint stiffness is a higher priority than re-tear risk.

## Introduction

Rotator cuff tears represent one of the most common causes of shoulder pain and dysfunction, particularly among middle-aged and elderly populations [1]. Advances in arthroscopic techniques have significantly improved visualization, tendon footprint reimplantation, and postoperative recovery [2]. However, the optimal timing for mobilization after rotator cuff repair remains controversial [3–5].

Early postoperative motion may prevent stiffness and facilitate early functional recovery, but it could threaten tendon-to-bone healing by increasing strain at the repair site [4, 6, 7]. Conversely, delayed mobilization promotes biologic healing of the tendon at the cost of transient stiffness [8]. Studies have shown that early rehabilitation can be safe for small tears, while medium and large tears may benefit from longer protection [9–11]. The development of knotless anchor systems has improved load distribution and reduced stress risers, potentially allowing for more controlled early motion. Nonetheless, biological healing capacity remains the critical determinant of repair success [12, 13]. Therefore, the primary objective of this study was to compare the clinical and structural outcomes of early versus delayed mobilization following arthroscopic single-row knotless rotator cuff repair for small and medium-sized tears. We hypothesized that while early mobilization would accelerate short-term range of motion recovery, delayed mobilization would result in superior structural tendon integrity with a lower re-tear rate, without compromising long-term functional and pain outcomes at one year.

## Materials and methods

### Study design and patients’ criteria

This prospective randomized study was conducted at Helwan University Hospitals from January 2024 to June 2025. In the current study we included age between 20 and 70 years, small or medium full-thickness supraspinatus tears (< 3 cm) confirmed by MRI, primary arthroscopic repair, intact subscapularis and absence of glenohumeral instability. However, we excluded from the study, massive tears (> 3 cm), revision surgery, significant glenohumeral arthritis or labral pathology and noncompliance with rehabilitation protocol. Patients were randomly assigned to one of two groups, EPM Group (*n* = 35): Passive motion initiated on postoperative day 1 and DPM Group (*n* = 35): Immobilization for 4 weeks before starting passive motion.

### Ethical statement

The study complied with the World Medical Association’s Declaration of Helsinki, according to the authors. This study was approved by the Faculty of Medicine, Helwan University Ethics Committee (Serial ID, 34-2026). All patients provided written informed consent prior to participation. The advantages, possible hazards, and every stage of the procedure were explained to all participants. Before taking part in the research, each participant signed an informed consent form.

### Sample size estimation

The sample size for this study was estimated based on a priori power analysis assuming a two-sided alpha error of 0.05, a statistical power of 80% (1-β), and an anticipated effect size derived from previous literature on rotator cuff rehabilitation, a minimum of 30 patients per group was required. To account for a potential dropout or loss to follow-up rate of approximately 15%, the sample size was adjusted to 35 patients per group, yielding a total of 70 enrolled patients. The sample size calculation was designed to detect clinically relevant differences in rehabilitation related functional outcomes and was not specifically powered to detect differences in the relatively infrequent event of tendon retear.

### Surgical technique

All surgeries were performed by a single surgeon in the beach-chair position under general anesthesia with interscalene block. Diagnostic arthroscopy and subacromial decompression were performed (when indicated). Tears were repaired using single-row knotless suture anchors, one anchor for small tears (< 1.5 cm) and two anchors for medium tears (1.5–3 cm).

The tendon footprint was anatomically restored with minimal tension. No abduction sling was used postoperatively.

### Rehabilitation protocol

**Fig. 1:**
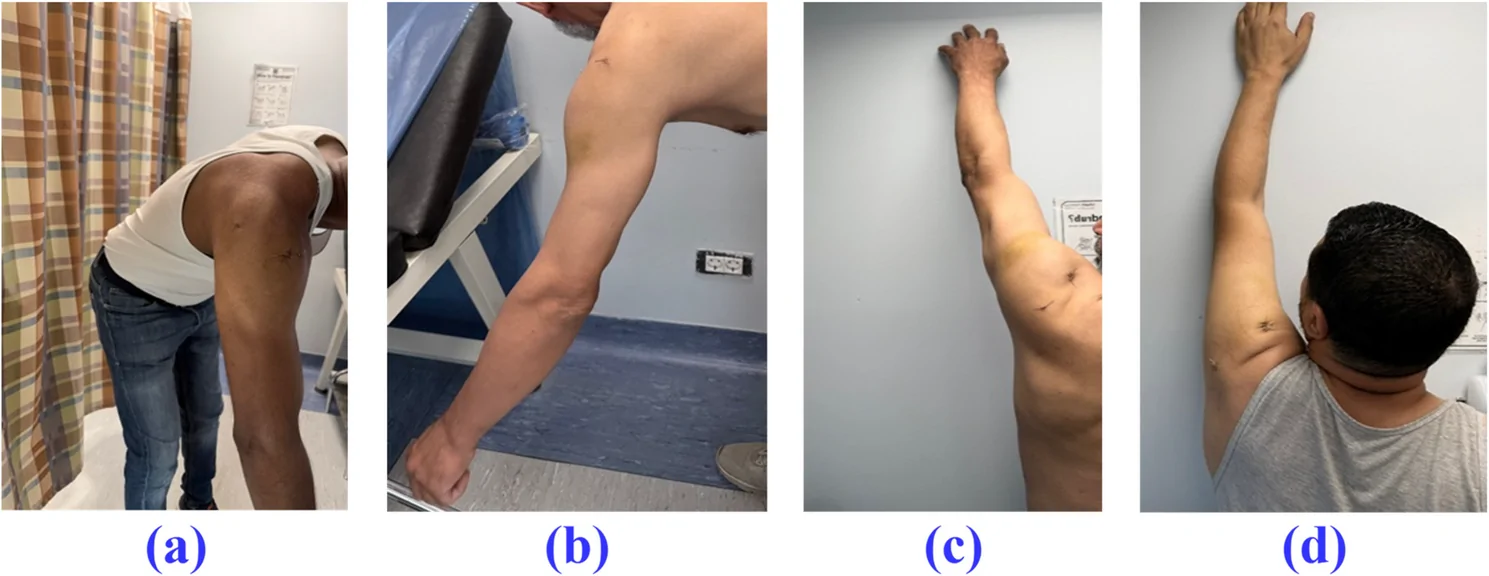
Passive pendulum and forward elevation exercises, **a**, **b**) pendulum exercises, and **c**, **d**) passive forward elevation

EPM: Passive pendulum and forward elevation exercises within 24 h; active-assisted motion after 4 weeks; strengthening at 8 weeks (Fig. 1). DPM: Immobilization for 4 weeks, followed by the same rehabilitation protocol (Fig. 1).

### All patients were subjected to

Clinical and demographic variables, including gender and age. The Sysmex KX-21 automated hematology analyzer (Sysmex Corporation, Japan) was used to get the entire blood picture, which included the differential leucocytic count. The Sysmex XT Automated Hematology Systems use hydrodynamic focusing and fluorescence flow cytometry. ALT, AST, total and direct bilirubin, serum albumin, and kidney function tests, including urea and creatinine levels: The Integra 400 auto-analyzer from Roche Diagnostics, Germany (COBAS INTEGRA^®^ 400 plus, 2009) was used to analyze all liver profile parameters. Hepatitis B and C virus screening in addition to abdominal ultrasound imaging.

### Outcome evaluation of the study

Assessments were performed at 6 weeks, 3, 6, and 12 months, included ROM: Forward flexion and external rotation using a goniometer. Pain: Visual Analog Scale (VAS, 0–10). Functional Scores: Constant–Murley and UCLA Shoulder Scores. MRI: Conducted at 12 months to evaluate tendon integrity using Sugaya classification.

### Statistical analysis methods

The data were collected, tabulated, and analyzed statistically using SPSS version 25 on a compatible IBM personal computer (Armonk, NY: IBM Corp.). Descriptive statistics refer to quantitative data presented in terms of median and range. The study utilized the Chi-squared test (χ2) to investigate the relationship between qualitative characteristics. When over 20% of the cells exhibit an expected count of less than 5, it is advisable to apply the Monte Carlo correction for the chi-square test or utilize Fisher’s Exact test. Two normally distributed datasets are compared through quantitative variables utilizing the student’s t-test (t). The Mann-Whitney U test (U) was employed to compare quantitative variables between two groups of non-normally distributed data. P-values below 0.05 are considered statistically significant.

## Results

**Fig. 2:**
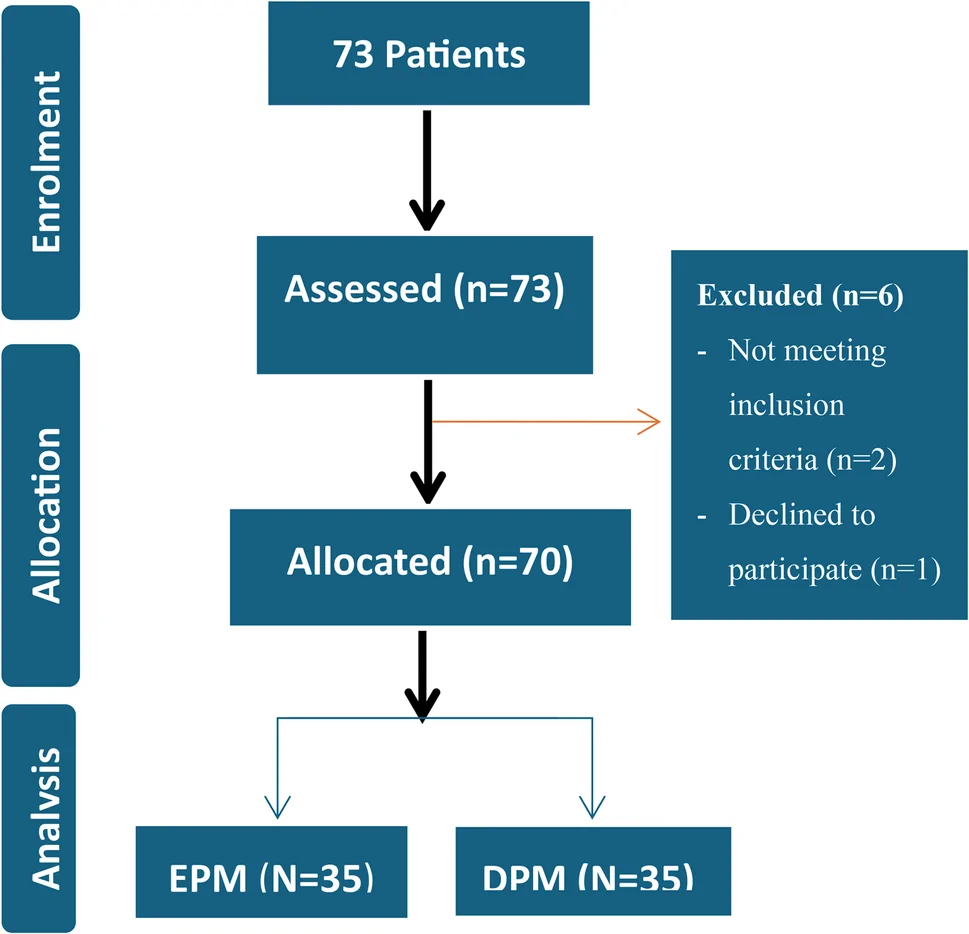
Flowchart of the patients studied

According to a workflow diagram that describes the cohort of 73 patients with small and medium-sized full-thickness supraspinatus tears who enrolled at Helwan University Hospitals, 3 individuals weren’t included in the study (1 denied consent and 2 did not match the inclusion criteria). This led to the inclusion of 70 patients in the research, divided into early mobilization (EPM, n = 35) and delayed mobilization (DPM, n = 35) (Fig. 2).

### Demographic data

Both groups were statistically comparable in baseline characteristics. The mean patient age was 56.8 ± 7.4 years in EPM and 57.3 ± 8.1 years in DPM (*p* = 0.78). Most patients presented with medium-sized tears repaired with two knotless anchors (Table 1).

**Table 1:** Demographic and baseline characteristics of the studied patients

Parameter	EPM (*n* = 35)	DPM (*n* = 35)	*p*-value
Age (years, mean ± SD)	56.8 ± 7.4	57.3 ± 8.1	0.78
Sex (M/F)	20/15	18/17	0.63
Dominant side involved (%)	60%	57%	0.81
Tear size (cm, mean ± SD)	2.1 ± 0.5	2.0 ± 0.6	0.67
Anchors used (1 / 2)	10 / 25	12 / 23	0.72

All data in this table were analysed using Chi-square test (X^2^), and Independent t-test (t)

### Functional and radiological outcomes

**Fig. 3:**
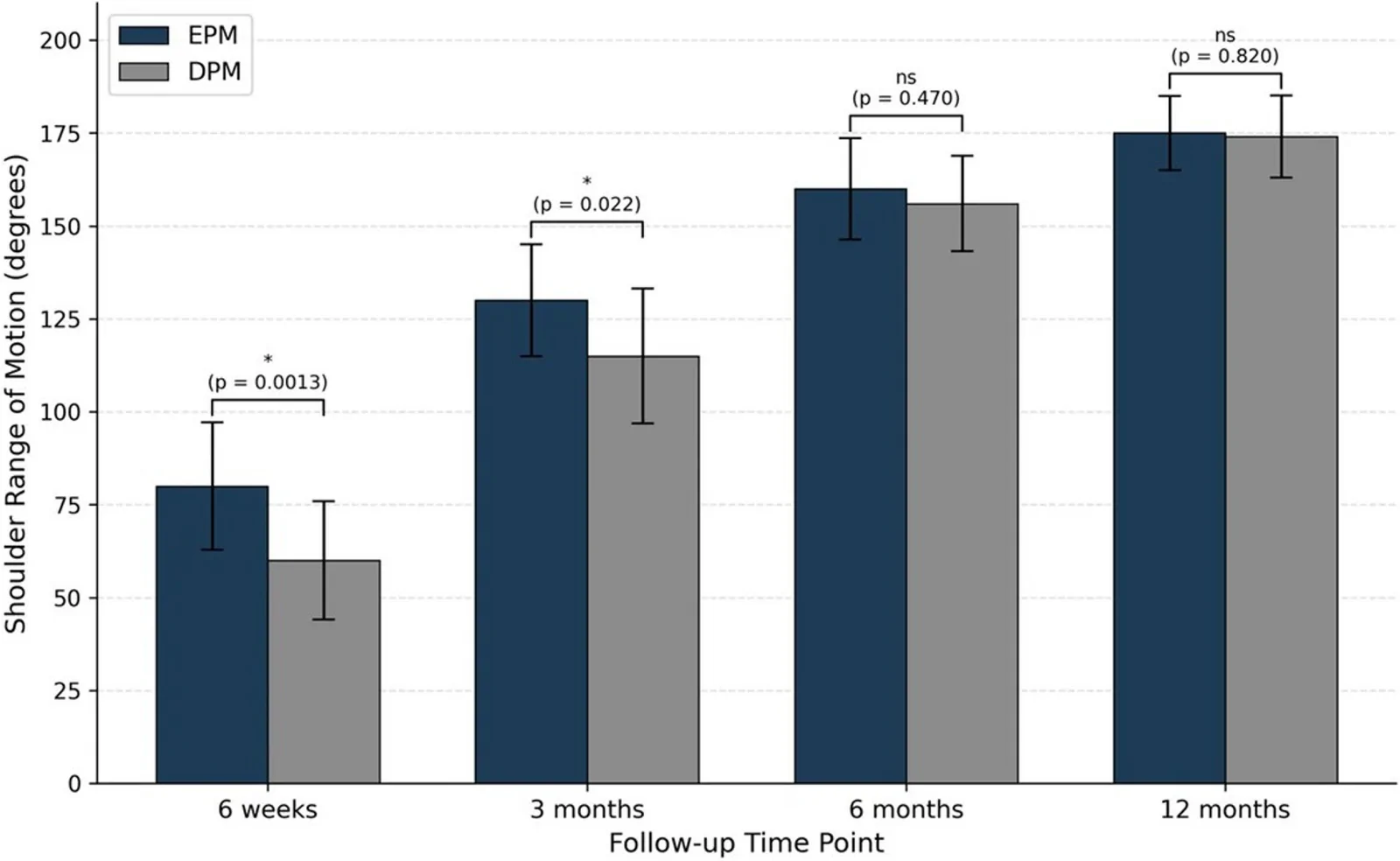
Comparison of shoulder ROM recovery (EPM vs. DPM)

At 3 months, the EPM group showed significantly greater ROM (130° ± 15°) compared to the DPM group (115° ± 18°, p = 0.02). However, by 12 months, both groups achieved similar functional outcomes (Table 2; Fig. 3).

**Table 2:** Functional outcomes among the studied patients

Outcome	EPM	DPM	*p*-value
Shoulder ROM recovery			
6 weeks	80 ± 17.13	60 ± 15.98	0.0013*
3 months	130 ± 15.11	115 ± 18.14	0.02 2*
6 months	160 ± 13.67	156 ± 12.85	0.470
12 months	175 ± 10.00	174 ± 11.05	0.820
Constant Score (12 months)	90 ± 6.00	89 ± 5.00	0.570
UCLA Score (12 months)	32 ± 2.05	31 ± 3.12	0.440
VAS Pain (12 months)	1.2 ± 0.5	1.3 ± 0.6	0.680

All data in this table were analysed using Mann-Whiteny U test (U), *Significant

### MRI Re-tear rate

EPM patients showed faster early recovery, but both groups converged by 12 months. At 12 months, MRI confirmed intact tendon repairs in 69 of 70 cases (98.6%). A single re-tear (2.8%) occurred in the EPM group in a medium-sized tear repaired with two anchors (Table 3). In the current study, no re-tears occurred in small tears repaired with a single anchor, supporting the mechanical adequacy of knotless single-row fixation for this tear size.

**Table 3:** MRI Re-tear rate (12 Months)

Group	Anchor Number	Re-tear Cases	% of Group
EPM	1 Anchor	1	2.8%
EPM	2 Anchors	0	0%
DPM	1–2 Anchors	0	0%

## Discussion

The superior short-term ROM observed in the EPM group at 6 weeks, and 3 months agrees with several recent meta-analyses. Hu et al. [14], pooling thirteen randomized controlled trials (RCTs) with 1,082 patients, reported that anterior flexion and abduction were significantly greater in EPM compared with DPM groups, concluding that early motion protocols were both safe and beneficial across tear sizes ranging from small to large. Similarly, in a randomized controlled trial of 206 patients, Sheps et al. [15] found that participants who self weaned from a sling and began early active motion had significantly better forward flexion and abduction than those managed with standard sling immobilization at 6 weeks postoperatively. Rhee et al. [16], studying patients undergoing biceps rerouting for large posterosuperior tears, likewise observed that the early motion group reached its maximal range of movement as early as 3 months, supporting the view that mobilizing the shoulder sooner accelerates the rate, though not necessarily the ceiling, of functional recovery. Mechanistically, this early benefit is generally attributed to reduced capsular adhesion formation, decreased fear-avoidance behaviour, and preservation of glenohumeral joint mobility during the critical early inflammatory phase of tendon healing, when immobilized shoulders are most prone to stiffness.

The loss of statistical significance between groups at 6 and 12 months in the present data mirrors a recurring finding in the literature: that early ROM gains are real but transient, with delayed rehabilitation groups largely catching up by mid to long term follow-up. Hu et al. [14] found no significant between group difference in external rotation, Constant-Murley score, or Simple Shoulder Test score, despite the early ROM advantage of EPM. Chen et al. [17], in a metanalysis of nine RCTs comprising 830 patients, similarly reported no discernible difference between early and delayed rehabilitation groups in pain, function, or healing rate at 6 and 12 months, and concluded that although one functional score differed statistically at 12 months, the magnitude fell below the threshold considered clinically meaningful. Rhee et al. [16] reported no clinically discernible difference in active ROM between early and delayed motion groups at 1-year follow-up despite the early plateau effect described above. Mazuquin et al. [18], in a systematic review and meta-analysis of twenty RCTs involving 1,841 patients, found no statistically significant differences in pain or function at almost any follow-up point between early and delayed groups, aside from an isolated improvement in patient-reported outcome at 6 months favouring early rehabilitation. Taken together, these findings support interpreting the present results as reflecting a genuine but time-limited early-mobilization advantage rather than a durable long-term superiority of EPM.

Not all studies concur with a straightforward benefit of early motion, and the literature reveals important disagreement, particularly regarding structural safety and the influence of tear size. Tirefort et al. [19], in a non-inferiority RCT, found no functional difference between three and six weeks of immobilization, arguing against the assumption that earlier mobilization is inherently superior and suggesting that a more conservative approach can be adopted without compromising outcomes. Gao et al. [20], comparing sling versus abduction brace immobilization strategies, similarly emphasized that the choice and duration of immobilization device did not consistently translate into superior functional outcomes, cautioning against over-generalizing the benefits of any single protocol. Hao et al. [21], reviewing RCTs comparing early exercise with brace fixation, echoed the concern raised in earlier work that unrestricted early loading could theoretically compromise tendon-to-bone healing in larger tears, even though their pooled analysis, like most others, did not detect a significant increase in retear rate. This tension reflects a long-standing debate in the shoulder rehabilitation literature: earlier meta-analyses had suggested that early ROM exercise could be associated with a higher risk of improper tendon healing specifically in large sized tears, whereas more recent, higher powered meta analyses including Hu et al. [14] and Chen et al. [17] have not confirmed a significant elevation in retear risk with early motion when passive, protected ranges are used. The present dataset does not report structural integrity or retear outcomes, and this remains an important limitation when interpreting the clinical significance of the early ROM advantage observed here.

From a clinical standpoint, the present findings support the use of EPM to accelerate early functional recovery and reduce the psychological and physical burden of prolonged immobilization, particularly during the first 6 to 12 postoperative weeks, when patients are most susceptible to stiffness, pain, and reduced quality of life. However, because the between group difference resolves by 6 months, clinicians should not conclude that DPM leads to inferior long-term outcomes; rather, the two approaches appear to differ chiefly in the rate, rather than the eventual extent, of ROM recovery. This is consistent with the broader literature on shoulder rehabilitation timing, including studies on total shoulder arthroplasty [22] and reverse total shoulder arthroplasty, which likewise report no consistent long-term advantage of early over delayed rehabilitation despite occasional early differences in range of movement or patient-reported outcomes. As with rotator cuff repair, tear size, tissue quality, and individual healing capacity likely moderate how much early motion can be safely tolerated, and rehabilitation protocols should continue to be individualized rather than uniformly applied.

Because the study was not powered for a tear size subgroup analysis, the findings should not be extrapolated to smaller tears. In the present cohort, the single retear occurred in a medium sized tear, while no retears were observed among the small tears; however, these observations are descriptive and should be interpreted cautiously.

### Limitations of the study

Several limitations should be acknowledged. First, the presented data report ROM in isolation, without accompanying functional scores, tendon integrity outcomes, or stratification by tear size, all of which are known to moderate the effect of rehabilitation timing in the literature reviewed above. Second, heterogeneity in the definition of early versus delayed motion across studies ranging from postoperative day 1 to 6 weeks limits direct comparability between the present findings and previously published protocols. Finally, as noted by several of the meta-analyses cited here, a substantial proportion of the underlying RCTs in this field carry an unclear or high risk of bias, underscoring the need for cautious interpretation and further high-quality, adequately powered trials stratified by tear size and repair technique. The study sample was adequate for the prespecified functional outcome analysis based on the a priori power calculation; however, it was not sufficiently powered to detect small differences in the incidence of tendon retear. Therefore, the observed difference of one retear versus none between groups should be considered descriptive rather than evidence of a statistically confirmed difference in healing.

## Conclusion

In conclusion, the findings support that early mobilization accelerates short-term ROM recovery, whereas both early and delayed protocols result in comparable functional and pain outcomes by 12 months. Given the limited number of retear events and the absence of a prespecified tear size subgroup analysis, no definitive recommendation can be made regarding a differential mobilization strategy according to tear size.

## Data Availability

All data supporting the study are presented in the manuscript or available upon request.
